# A modular interface of IL-4 allows for scalable affinity without affecting specificity for the IL-4 receptor

**DOI:** 10.1186/1741-7007-4-13

**Published:** 2006-04-26

**Authors:** Michael Kraich, Markus Klein, Edwin Patiño, Henning Harrer, Joachim Nickel, Walter Sebald, Thomas D Mueller

**Affiliations:** 1 Lehrstuhl für Physiologische Chemie II, Theodor-Boveri Institut für Biowissenschaften (Biozentrum) der Universität Würzburg, Am Hubland, D-97074 Würzburg, Germany; 2 Rudolf-Virchow Zentrum, DFG Forschungszentrum für Experimentelle Biomedizin, Versbacher Str. 9, D-97078 Würzburg, Germany

## Abstract

**Background:**

Interleukin 4 (IL-4) is a key regulator of the immune system and an important factor in the development of allergic hypersensitivity. Together with interleukin 13 (IL-13), IL-4 plays an important role in exacerbating allergic and asthmatic symptoms. For signal transduction, both cytokines can utilise the same receptor, consisting of the IL-4Rα and the IL-13Rα1 chain, offering an explanation for their overlapping biological functions. Since both cytokine ligands share only moderate similarity on the amino acid sequence level, molecular recognition of the ligands by both receptor subunits is of great interest. IL-4 and IL-13 are interesting targets for allergy and asthma therapies. Knowledge of the binding mechanism will be important for the generation of either IL-4 or IL-13 specific drugs.

**Results:**

We present a structure/function analysis of the IL-4 ligand-receptor interaction. Structural determination of a number of IL-4 variants together with *in vitro *binding studies show that IL-4 and its high-affinity receptor subunit IL-4Rα interact via a modular protein-protein interface consisting of three independently-acting interaction clusters. For high-affinity binding of wild-type IL-4 to its receptor IL-4Rα, only two of these clusters (*i.e*. cluster 1 centered around Glu9 and cluster 2 around Arg88) contribute significantly to the free binding energy. Mutating residues Thr13 or Phe82 located in cluster 3 to aspartate results in super-agonistic IL-4 variants. All three clusters are fully engaged in these variants, generating a three-fold higher binding affinity for IL-4Rα. Mutagenesis studies reveal that IL-13 utilizes the same main binding determinants, *i.e*. Glu11 (cluster 1) and Arg64 (cluster 2), suggesting that IL-13 also uses this modular protein interface architecture.

**Conclusion:**

The modular architecture of the IL-4-IL-4Rα interface suggests a possible mechanism by which proteins might be able to generate binding affinity and specificity independently. So far, affinity and specificity are often considered to co-vary, *i.e*. high specificity requires high affinity and vice versa. Although the binding affinities of IL-4 and IL-13 to IL-4Rα differ by a factor of more than 1000, the specificity remains high because the receptor subunit IL-4Rα binds exclusively to IL-4 and IL-13. An interface formed by several interaction clusters/binding hot-spots allows for a broad range of affinities by selecting how many of these interaction clusters will contribute to the overall binding free energy. Understanding how proteins generate affinity and specificity is essential as more and more growth factor receptor families show promiscuous binding to their respective ligands. This limited specificity is, however, not accompanied by low binding affinities.

## Background

Interleukin 4 (IL-4) is a pleiotropic cytokine that plays a major regulatory role in the immune system [1]. IL-4 induces the differentiation of T-helper cells to a type 2 (T_H_2) cytokine-producing phenotype [2] and the class switching to IgE and IgG4 [3,4]. Furthermore, it stimulates the expression of adhesion molecules such as VCAM-1 [5] and chemokines such as eotaxin-1, -2 and -3 [6-8]. Activated T_H_2 cells play a triggering role in the activation and/or recruitment of IgE antibody-producing B cells, mast cells [9] and eosinophils [10], which are all involved in allergic inflammation [11]. Therefore, IL-4 plays a key role in the development and progression of allergic hypersensitivity.

**Figure 1:**
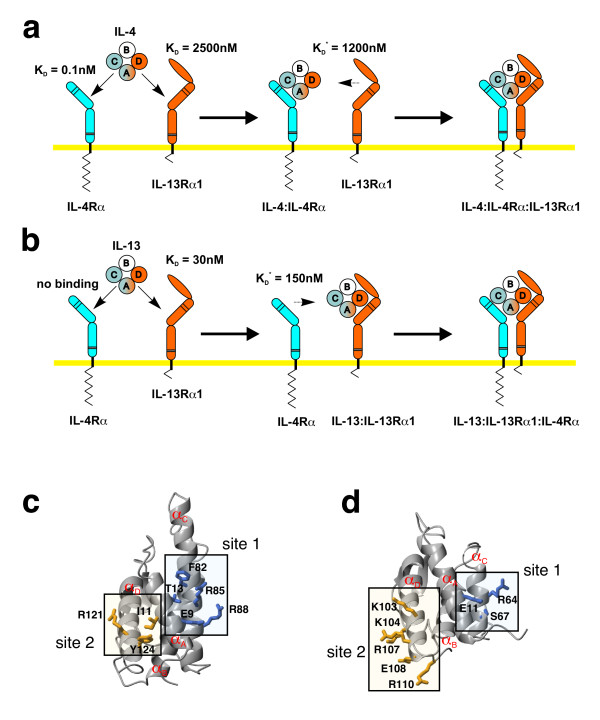
**Sequential binding mechanism in the IL-4/-13 receptor activation**. (a) The binding of IL-4 to its cellular receptor follows a two-step sequential binding mechanism. First, IL-4 is recruited to the membrane by its high-affinity subunit IL-4Rα; second, either one of the two low affinity subunits IL-13Rα1 (apparent *K*_D _~ 1 μM) or γ_c _(app. *K*_D _~ 1.5 – 2 μM) is recruited into the complex. (b) For IL-13 the order of the binding events is reversed. IL-13 binds first to the IL-13Rα1 subunit; the affinity of IL-13 to IL-4Rα is below detection limit (app. *K*_D _> 100 μM). In the second step the IL-13:IL-13Rα1 complex recruits the IL-4Rα subunit into the complex. Values marked *K*_D_* indicate that these interactions are measured by binding of the soluble ectodomain to the surface immobilized binary complex of ligand and high-affinity receptor subunits. These apparent binding constants do not reflect the real affinity for a two-dimensional interaction in the membrane. (c, d) The location of the binding sites for the receptor subunits IL-4Rα and IL-13Rα1 are conserved between the two cytokines IL-4 (c) and IL-13 (d). Site 1 is used for the interaction with the IL-4Rα subunit, site 2 is used for the interaction with IL-13Rα1 (and for binding to γ_c _in the case of IL-4).

Signal transduction of IL-4 is mediated by a sequential binding process, initiated first by binding of IL-4 to its high-affinity single membrane spanning receptor chain IL-4Rα (Fig. 1a). This intermediate ligand receptor complex then recruits one of two possible low-affinity receptor subunits, the common gamma chain (γ_c_) [12,13] or the IL-13Rα1 chain [14,15], into the functional hetero-trimeric complex to initiate signalling. The γ_c _receptor subunit is shared among the cytokines IL-2, -4, -7, -9, -15 and -21 [12,13], whereas the IL-13Rα1 subunit is exclusively used by IL-4 and -13 [16].

IL-13 shares only 25% identity with IL-4 on the amino acid sequence level [17]. Despite this moderate homology, IL-13 and IL-4 utilize an identical cellular receptor built from the subunits IL-4Rα and IL-13Rα1 (Fig. 1b) [16]. However, the order of the binding sequence and binding affinities to the individual receptor subunits differ markedly between the two cytokines. In contrast to IL-4, IL-13 binds first to the IL-13Rα1 subunit with high affinity and subsequently recruits the IL-4Rα chain as the low-affinity receptor subunit into the complex. High-affinity binding of IL-4 to its cellular receptor is mediated almost exclusively by the IL4-Rα subunit (Fig. 1a) [18]. The binding of IL-4 to the extracellular domain of IL-4Rα determined by surface plasmon resonance spectroscopy yields a dissociation constant *K*_D _of approx. 0.1 – 0.2 nM [19]. In the case of IL-4, the low-affinity receptor subunits IL-13Rα1 and γ_c _[20] seem to contribute little to the overall binding affinity (Fig. 1a). For IL-13, only binding to its high-affinity subunit has been determined *in vitro *so far (*K*_D _~ 25 – 50 nM) [21], and this is confirmed by binding experiments using CHO cells transfected with IL-13Rα1 (*K*_D _~ 4 – 5 nM). Binding to the receptor formed from IL-13Rα1 and IL-4Rα leads to a dramatic increase in affinity (*K*_D _~ 30 – 40 pM), suggesting a strong cooperativity for binding to both receptor subunits (Fig. 1b) [15].

The structures of both cytokine ligands IL-4 and IL-13 have been determined by x-ray crystallography or by NMR [22-27]. Both IL-4 and IL-13 belong to the short-chain 4-helix bundle cytokines. The four anti-parallel helices A, C, B and D are connected by two long end-to-end loops AB and CD and one short loop connecting helices B and C (Fig. 1c, d). Structure-function analyses of IL-4 have revealed insights into the architecture and composition of the binding epitopes involved in the binding to the IL-4Rα and the γ_c _receptor subunits [18-20], [28-32]. Structure determination of the ligand-receptor complex of IL-4 bound to its high-affinity receptor subunit IL-4Rα has shown that the protein-protein interface represents a new type of modular architecture [33]. Instead of a continuous binding epitope, three so-called interaction clusters could be identified, which contribute affinity independently and might therefore allow for a scalable affinity to different cytokine ligands [34]. Interestingly, the location of the binding sites for the receptor subunits IL-4Rα and IL-13Rα1 are conserved in both ligands IL-4 and IL-13, as shown by mutagenesis studies [35-37] and structural analyses, suggesting that similar residues might be involved in the recognition and binding of these receptor subunits. In this paper we explore the possibility that this new architecture is the basis of the high specificity of the IL-4Rα subunit for the two cytokines IL-4 and IL-13 and its simultaneous variable binding affinity to both ligand proteins. The results contribute significantly to the understanding of how proteins might generate binding affinity and specificity independently, allowing promiscuous protein-protein interactions.

## Results

### Two mutations in helix C convert IL-4 into a super-agonist

**Figure 2:**
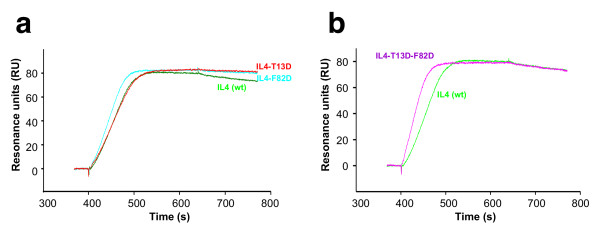
**BIAcore analysis of super-agonistic IL-4 variants**. BIAcore sensorgrams of ligand receptor interactions. Solutions of the indicated IL-4 variants (concentration 10 nM) were perfused over a sensor chip with immobilized IL4Rα_ECD_. (a) Compared to wild-type IL-4 (green) the super-agonistic variants IL4-T13D (red) and IL4-F82D (cyan) show higher binding-affinities to IL4Rα_ECD _owing to an approximately 3 times slower dissociation rate *k*_off_. (b) The double variant IL4-T13D-F82D (purple) shows no altered binding-affinity to IL4Rα_ECD _in comparison to wild-type IL-4 (green).

By mutational analysis, two IL-4 variants were found with higher binding affinity to IL-4Rα than wild-type IL-4 [30]. The binding characteristics were analyzed by biosensor-based experiments. An IL-4Rα variant (extracellular domain comprising residues 1 to 210), which harbours the mutations C182A and Q207C (IL-4Rα_ECD_), was used for BIAcore studies allowing the receptor protein to be immobilized in an oriented fashion. The receptor protein was biotinylated via the free thiol group and immobilized on a streptavidin-coated BIAcore CM5 biosensor chip. Analysis of the variants T13D and F82D yielded dissociation constants *K*_D _of 0.02 – 0.04 nM and 0.03 – 0.04 nM respectively (Table 1). These values are 2- to 3-fold lower than those for the interaction between wild-type IL-4 and IL-4Rα (Table 1). The increase in affinity is mainly attributable to the reduced dissociation rates (*k*_off_) of T13D and F82D. Wild-type IL-4 dissociates from the immobilized extracellular domain of IL4Rα (IL-4Rα_ECD_) with a rate of 1.3 × 10^-3^s^-1^, which is approximately 2–3 times faster than for T13D or F82D (Fig. 2a; Table 1). The association rates (*k*_on_) seem not to be affected by these mutations (Table 1). Therefore, the introduction of an acidic residue does not alter the electrostatic steering effect observed for the IL-4-IL-4Rα interaction. Since it is mainly the dissociation process that is altered in the T13D and F82D variants, it can be concluded that an acidic residue at either position 13 or 82 leads to a stabilization of the IL-4-IL4-Rα_ECD _complex, resulting in IL-4 super-agonists. This indicates that additional non-covalent interactions between the ligand and the receptor ectodomain must be formed.

**Table 1:** BIAcore analysis of IL-4, IL-13 and variants

IL-4 variant	*k*_on _× 10^-7 ^[s^-1 ^M^-1^]	*k*_off _× 10^3 ^[s^-1^]	app. *K*_D _[nM]	relative *K*_D _(*K*_D_(mut)/*K*_D_(IL-4))
IL4	1.32 ± 0.27	1.26 ± 0.16	0.10 ± 0.02	1.0
T13D	1.27 ± 0.19	0.46 ± 0.16	0.04 ± 0.02	0.4
F82D	1.61 ± 0.15	0.46 ± 0.15	0.03 ± 0.01	0.3
T13D-F82D	1.19 ± 0.18	1.40 ± 0.27	0.12 ± 0.03	1.2
R85A	0.46 ± 0.22	1.58 ± 0.17	0.47 ± 0.34	4.7
T13D-R85A	0.58 ± 0.19	1.38 ± 0.22	0.26 ± 0.08	2.6
F82D-R85A	0.42 ± 0.12	4.10 ± 1.01	1.08 ± 0.50	10.8
T13D-F82D-R85A	0.30 ± 0.08	1.44 ± 0.19	0.51 ± 012	5.1

IL-13 Variant				relative *K*_D _(*K*_D_(mut)/*K*_D_(IL-13))

IL-13	-	-	-	1.0 (150 nM)
E11A	-	-	-	233
R64A	-	-	-	> 1300

Association and dissociation rates of IL-4 variants to immobilized IL4-Rα_ECD _were measured on a BIA2000 system. The rate constants represent mean values of 18 independent measurements with 6 different analyte concentrations. Binding affinities of IL-4Rα_ECD _to the complex of IL-13/IL-13variant bound to IL-13Rα1 were measured via the COINJECT command. Dissociation constants were obtained from equilibrium binding analysis, therefore no rate constants are given. Binding of the IL-13 variants to IL-13Rα1 was unaltered compared to wild-type IL-13.

To determine whether the stabilizing effects of the two single mutations T13D and F82D might act in a cooperative manner, we generated the IL-4 double variant T13D-F82D, which we expected to bind IL-4Rα with a *K*_D _in the sub-picomolar range. However, interaction analysis revealed that the apparent *K*_D _of the complex between IL-4Rα and this IL-4 variant is higher than that observed for the individual single mutation variants T13D and F82D and is similar to that for wild-type IL-4 (Fig. 2b; Table 1). In addition, the kinetic rate constants, especially the dissociation rate, clearly reflect the binding characteristics of wild-type IL-4 (Fig. 2b). The two amino acid exchanges therefore seem not to act cooperatively; the additional thermodynamic stabilization of T13D and F82D leads to competition, possibly because both variants have identical binding mechanisms. To test whether binding specificity is altered by these mutations, binding to other cytokine receptor subunits was compared with that of wild-type IL-4. Binding to the low-affinity subunits was determined by measuring the affinities of IL-4, T13D and F82D bound to IL-4Rα for IL-13Rα1 and γ_c_. Direct binding of IL-4 and the super-agonistic variants to the low-affinity subunit IL-13Rα1 was also measured (IL-4: *K*_D _= 2.5 μM; T13D: *K*_D _= 2.5 μM; F82D: *K*_D _= 2.3 μM). The affinity of direct interaction between IL-4 (and T13D as well as F82D) and γ_c _is too low (*K*_D >_ 100 μM) to be detected by BIAcore technology. No differences between wild-type IL-4 and the super-agonist variants could be observed; the affinity for IL-13Rα1 is similar for all three binary ligand receptor complexes (*K*_D _= 1.2 μM IL-4wt:IL-4Rα; *K*_D _= 1.7 μM T13D:IL-4Rα; *K*_D _= 1.2 μM F82D:IL-4Rα). Similar observations were made for the interaction of IL-4 wild-type and super-agonist proteins with the low-affinity subunit γ_c _(IL-4:IL-4Rα : *K*_D _= 1.7 μM; T13D:IL-4Rα : *K*_D _= 2 μM; F82D:IL-4Rα : *K*_D _= 1.2 μM). This result indicates that the mutations at positions 13 and 82 in the IL-4Rα binding site (site 1) of IL-4 do not alter interactions at the binding site for the two low-affinity receptor subunits IL-13Rα1 and γ_c _(Fig. 1c, d). In addition, the cytokine receptor IL-21R, which shares the highest amino acid sequence similarity with IL-4Rα in the extracellular part, was used as a control for specificity. Neither wild-type IL-4 nor the super-agonistic variants T13D and F82D showed any binding to this receptor subunit.

### Structural analysis of super-agonist variants

**Figure 3:**
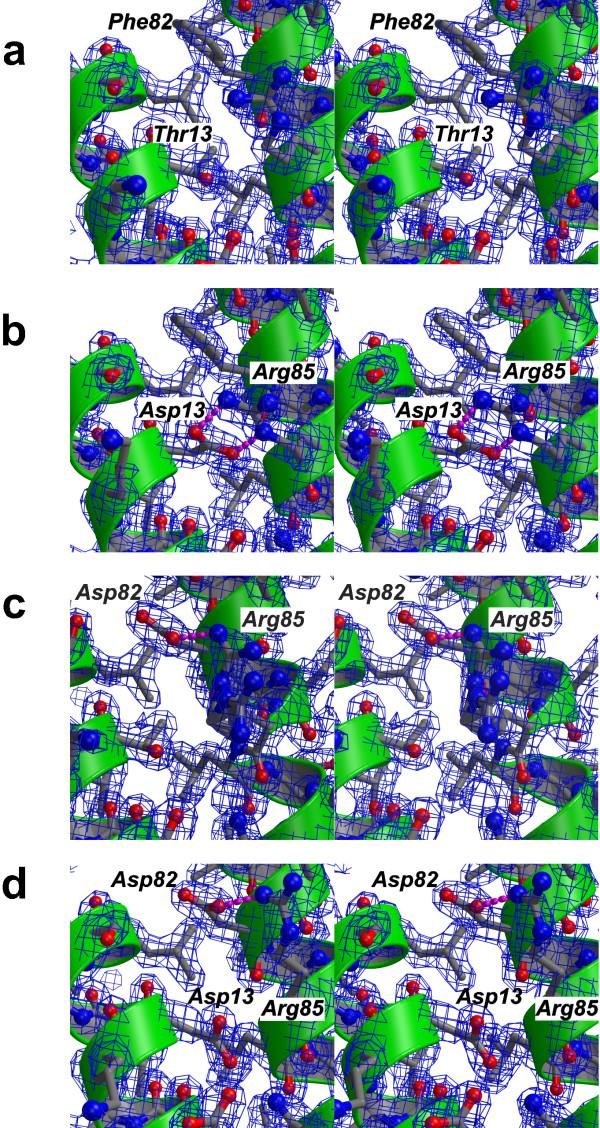
**Small structural changes probably account for super-agonist binding properties**. Figures a-d are presented as wall-eyed stereo images. (a) Magnification (stereo image) of the region around Thr13 and Phe82 of wild type IL-4, a 2*F*_obs _– *F*_calc _electron density map is shown at a level of 1.5σ. (b) The same area is shown for the super-agonistic IL-4 variant T13D; the exchange of Thr13 for an aspartate leads to a change in side chain conformation of Arg85, which exhibits a bi-dentate hydrogen bond between the carboxylate group of Asp13 and the guanidinium group of Arg85. (c) Area shown for the super-agonist IL-4 F82D; as in (b) a hydrogen bond between Asp82 and Arg85 leads to a change in side chain conformation of Arg85. However, two alternative side chain conformations can be observed for Arg85, one that is "bound" to Asp82 and a second where the Arg85 side chain is oriented towards the solvent. (d) The double variant IL-4 T13D/F82D shows similar side chain orientations to those in IL-4 F82D, but only a single side chain conformation is observed for Arg85.

To elucidate the molecular mechanism by which the two mutations might lead to additional stabilizing interactions, we determined the high-resolution structures of the IL-4 variants T13D and F82D as well as the double variant T13D-F82D. Improvements in the purification procedure, especially the use of fractional ammonium sulfate precipitation steps, enabled us to obtain highly homogenous and pure IL-4 protein, giving a single protein band on silver-stained SDS polyacrylamide gels. The high homogeneity of the protein enabled large crystals (approx. 1.2 × 0.3 × 0.3 mm) to be prepared, which diffracted to high resolution. The structures of the IL-4 variants T13D (max. resolution 1.65 Å), F82D (max. resolution 1.7Å) and T13D-F82D (max. resolution 1.8Å) were refined on the basis of the structure of wild-type IL-4 (max. resolution 1.8Å); all crystals were obtained under identical conditions, allowing detailed comparison. The overall structure of IL-4 comprises the known four-helical bundle in up-up-down-down topology (Fig. 1c) [24,26,27,31,38], which like the crystal packing is unaffected by these mutations. The r.m.s. deviation for the protein backbone is less than 0.5Å. The only differences that can be observed between the structures of IL-4 (Fig. 3a) and these three variants (Figs. 3b-d) are located in very close proximity to the site of mutation.

In the variant T13D, the aspartate residue resides in the middle of the first α-helix α_A_, and the side chain is oriented towards α-helix C. Both carboxylate oxygens of Asp13 are involved in a bi-dentate salt bridge to Arg85 on helix α_c_, thereby tightly fixing the side chain of Arg85 (Fig. 3b). The geometries and distances of the bi-dentate hydrogen bonds are close to ideal parameters; the planes formed by the carboxylate of Asp13 and the guanidinium group of Arg85 are out of planarity by just 14°; the lengths of the two hydrogen bonds are 2.7 and 2.8Å. For comparison, in the structure of wild-type IL-4, the hydroxyl group of Thr13 is hydrogen-bonded to the main chain carbonyl of Glu9; the side chain of Arg85 is moved outward towards the solvent and fixed to the hydroxyl group of Thr13 by a water-mediated H-bond. The change in side chain conformation of Arg85 becomes clear if the side chain torsion angles of the wild-type and variant are compared. The χ_1 _torsion angle of Arg85 is in the trans conformation (174° IL-4, 176° IL-4 T13D) for both proteins; in the case of IL-4, χ_2 _of Arg85 is slightly off trans (160°), whereas for T13D, the torsion angle χ_2 _of Arg85 is in the trans conformation (176°). In contrast, the χ_3 _torsion angle differs by 115° between IL-4 (58°, gauche^- ^conformation) and T13D (-57°, gauche^+ ^conformation). In the lower resolution structures of IL-4 (PDB entries 1RCB, 1HIK) the temperature factors for the side chain atoms of Arg85 were elevated compared to the main chain atoms, indicating greater side chain flexibility. In contrast, in our current study, the temperature factors for the side chain atoms of Arg85 are of similar magnitude in the high-resolution structures of wild-type IL-4 (mean 23Å^2 ^for side chain from C_β_) and its variant T13D (mean 20Å^2^). These values are also close to those of the atoms located in the hydrophobic core (mean 15Å^2^), therefore the side chain of Arg85 can be considered rigid.

The structure of the super-agonist variant F82D shows a different picture (Fig. 3c); the side chain conformation, i.e. the χ_1 _and χ_2 _torsion angles, of Asp82 is identical to that of the wild-type Phe82. As in IL-4 T13D, the only structural changes are close to the site of mutation. Again the orientation of the side chain of Arg85 is changed from that in IL-4, but the side chain conformations of Arg85 are different in T13D and F82D. Interestingly, the high-resolution structure of variant F82D reveals two alternative side chain conformations for Arg85. In one, the side chain is not hydrogen-bonded to the Asp residue introduced at position 82 (Fig. 3c) and is oriented towards the solvent. In the second, the side chain interacts with the carboxylate group of Asp82 via a weak single hydrogen bond, but it is much less fixed than in the variant T13D (Fig. 3b). The distance between the amino group of Arg85 and the carboxylate group of Asp82 is 3.2Å and therefore close to the exclusion criterion for a hydrogen bond. In addition, the temperature factors of the Arg85 side chain atoms are elevated compared to those of rigid main chain or side chain atoms in the variant F82D, indicating that the stabilization of a certain side chain conformation is not as rigid as in variant T13D.

In the case of the double variant, T13D-F82D, the side chain of Arg85 also occupies a different side chain conformation from that in wild-type IL-4. Although two "acceptor" carboxylate groups are present, Asp13 and Asp82, the side chain of Arg85 exhibits only one defined side chain conformation in which the guanidinium group is hydrogen bonded to the carboxylate group of Asp82 (Fig. 3d). As in variant F82D, the temperature factors of the side chain atoms of Arg85 are elevated compared to main chain atoms. The fixation of the side chain of Arg85 to the core structure of IL-4 might therefore be not as strong as observed for T13D. Although only a single defined side chain conformation for Arg85 is observed in T13D-F82D, the loss in affinity of the double variant compared to both super-agonists might result from a competition between two possible side chain conformations of Arg85.

### Interaction analysis using IL-4 receptor variants

The binding behaviour of the super-agonists suggests that additional interactions are formed between the ligand and the IL-4 receptor α-chain. To determine whether these interactions are formed directly between the substituted side chains of T13D or F82D or result from indirect interaction, we used variants of IL-4Rα to identify the potential interaction partners of Asp13 (T13D) and Asp82 (F82D). Single amino acid variants of IL-4Rα_ECD _were immobilized on a BIAcore CM5 biosensor chip similar to the one described above. Four IL-4Rα variants were chosen on the basis of the location of the respective side chain in the ligand-receptor interface. The IL-4Rα variant Y13F was selected as a control because the hydroxyl group of Tyr13 is located far from the site of mutation of the two super-agonists T13D and F82D (distance: Asp13 or Phe82 side chain of T13D and F82D – IL-4Rα Tyr13 = 10Å). Therefore, the effect of the IL-4Rα Y13F mutation on the affinity for ligand should be identical in magnitude for wild-type IL-4 and both super-agonistic variants T13D and F82D. In fact, the affinity of IL-4Rα Y13F to wild-type IL-4 is reduced by a factor of ~ 20 compared to wild-type IL-4Rα. The affinities for the super-agonists T13D and F82D are decreased similarly by factors of 33 and 19, respectively (Table 3).

**Table 2:** Processing and refinement statistics for IL-4 and variants

Crystal	IL-4 WT	IL-4T13D	IL-4F82D	IL-4 T13DF82D	IL-4R85A	IL-4 T13DR85A	IL-4 F82DR85A
Spacegroup	P4_1_2_1_2						
Beamline	ESRF ID14	SLS XS06	SLS XS06	ESRF ID14	Rigaku MicroMax007, VariMaxCu HighRes		
Wavelength	0.9500	0.9178	0.9183	0.9500	1.5418	1.5418	1.5418
Unit Cell (Å)	a = b = 90.54, c = 46.07	a = b = 90.53, c = 45.91	a = b = 90.97, c = 46.08	a = b = 91.03, c = 46.02	a = b = 91.13, c = 45.82	a = b = 91.33, c = 45.78	a = b = 91.20, c = 46.00
Resolution (Å)	20.5 – 1.8 (1.9 – 1.8)	10.6 – 1.65(1.74 – 1.65)	28.7 – 1.6 (1.69 – 1.6)	20.5 – 1.8 (1.9 – 1.8)	32.3 – 2.35 (2.43 – 2.35)	45.7 – 2.1 (2.18 – 2.1)	26.4 – 2.0 (2.07 – 2.0)
Total Reflections	94871 (18941)	152584 (40910)	375353 (34716)	104168 (24465)	36412 (3611)	60477 (6012)	70758 (6893)
Completeness (%)	99.0 (98.5)	97.1 (98.1)	100.0 (99.9)	99.6 (100.0)	98.9 (99.6)	99.4 (98.7)	98.9 (98.2)
Multiplicity	5.2 (3.6)	6.7 (6.4)	14.4 (9.3)	5.7 (4.7)	4.35 (4.32)	5.16 (5.15)	5.26 (5.29)
R_merge_	0.058 (0.230)	0.092 (0.172)	0.080 (0.291)	0.071 (0.211)	0.056 (0.265)	0.055 (0.258)	0.050 (0.278)
Average I/sigma	18.6 (4.6)	17.6 (5.2)	26.6 (5.7)	15.2 (4.9)	17.3 (4.6)	18.2 (5.6)	19.2 (5.2)

Refinement							

Resolution (outer shell) (Å)	14.9 – 1.8 (1.85 – 1.8)	10.0 – 1.65 (1.69 – 1.65)	20.0 – 1.7 (1.74 – 1.7)	14.8 – 1.8 (1.85 – 1.8)	20.0 – 2.50 (2.56 – 2.50)	20.0 – 2.1 (2.15 – 2.1)	20.0 – 2.0 (2.05 – 2.0)
# of reflections/in test set	17121/926 (1225/74)	21555/1142 (1533/84)	20694/1100 (1492/77)	17414/942 (1248/70)	6654/334 (472/28)	11162/555 (818/34)	12767/664 (893/55)
R factor	0.211 (0.219)	0.221 (0.253)	0.226 (0.246)	0.218 (0.263)	0.214 (0.224)	0.214 (0.250)	0.205 (0.253)
Free R factor	0.244 (0.272)	0.247 (0.319)	0.248 (0.281)	0.241 (0.303)	0.264 (0.316)	0.249 (0.337)	0.258 (0.308)
Average B factor (Å^2^)	27.2	21.1	34.8	26.4	34.5	22.1	28.8
R.m.s.d. Bonds	0.017	0.018	0.011	0.011	0.012	0.016	0.014
R.m.s.d. Angles	1.597	1.695	1.322	1.194	1.400	1.661	1.379

Ramachandran							

Most favored	89.4	91.9	89.4	91.9	91.1	92.7	92.7
Additionally favored	9.8	8.1	10.6	7.3	8.9	7.3	7.3
Generously or disallowed	0.8 (1, Thr22)	-	-	0.8 (1, Lys37)	-	-	-

**Table 3:** BIAcore analysis of IL-4Rα variants

	IL-4			IL4-T13D		
IL4-Rα variant	*k*_on _× 10^-7 ^[s^-1 ^M^-1^]	*k*_off _× 10^3 ^[s^-1^]	app. *K*_D _[nM]	*k*_on _× 10^-7 ^[s^-1 ^M^-1^]	*k*_off _× 10^3 ^[s^-1^]	app. *K*_D _[nM]

IL4-Rα	1.79 ± 0.27	1.62 ± 0.67	0.09 ± 0.04	1.24 ± 0.20	0.25 ± 0.20	0.02 ± 0.02
Y13T	1.55 ± 0.67	23.5 ± 4.05	1.76 ± 0.75	1.03 ± 0.43	5.38 ± 0.51	0.66 ± 0.43
D67A	0.80 ± 0.34	36.5 ± 7.60	5.60 ± 3.32	0.52 ± 0.19	1.82 ± 0.39	0.38 ± 0.10
D125A	0.89 ± 0.31	6.46 ± 1.75	0.77 ± 0.23	0.79 ± 0.14	0.65 ± 0.29	0.08 ± 0.04
Y127F	1.17 ± 0.51	1.99 ± 0.65	0.20 ± 0.07	0.96 ± 0.15	0.91 ± 0.34	0.09 ± 0.02

	IL4-F82D			IL4-T13D-F82D		

IL4-Rα variant	*k*_on _× 10^-7 ^[s^-1 ^M^-1^]	*k*_off _× 10^3 ^[s^-1^]	app. *K*_D _[nM]	*k*_on _× 10^-7 ^[s^-1 ^M^-1^]	*k*_off _× 10^3 ^[s^-1^]	app. *K*_D _[nM]
IL4-Rα	1.56 ± 0.93	0.48 ± 0.37	0.04 ± 0.04	1.06 ± 0.26	1.44 ± 0.96	0.14 ± 0.11
Y13T	1.20 ± 0.36	8.64 ± 1.74	0.76 ± 0.21	0.68 ± 0.32	31.8 ± 3.42	4.22 ± 0.62
D67A	0.68 ± 0.18	8.82 ± 1.83	1.37 ± 0.47	0.52 ± 0.11	8.56 ± 1.63	1.67 ± 0.27
D125A	0.99 ± 0.22	2.02 ± 0.39	0.21 ± 0.06	0.55 ± 0.11	1.34 ± 0.40	0.24 ± 0.06
Y127F	1.12 ± 0.21	1.08 ± 0.33	0.10 ± 0.03	0.40 ± 0.24	27.4 ± 6.01	6.15 ± 2.68

Association and dissociation rates of IL-4 variants to immobilized IL4-Rα_ECD _variants were measured on a BIA2000 system. The rate constants represent mean values of 12 independent measurements with 6 different analyte concentrations.

The other three IL-4Rα variants either carry mutations close to the interacting site or the substituted residues interact directly with the side chains of Thr13 or Phe82 in the ligand. The carboxylate group of IL-4Rα Asp67 forms a bi-dentate hydrogen bond with the guanidinium group of IL-4 Arg85, therefore mutation of IL-4Rα Asp67 to Ala markedly decreases the binding affinity (IL-4Rα D67A : IL-4 ~ 60-fold). In comparison, the affinity of IL-4Rα D67A for the super-agonist T13D is only reduced 19-fold, compared to 34-fold for F82D. Aspartate 125 of IL-4Rα interacts with the side chains Gln78 and Arg81 of IL-4 and is in close proximity to residue Phe82 of IL-4. Mutation of Asp125 to alanine in IL-4Rα reduces the binding affinity to IL-4 8-fold, owing to the loss of two hydrogen bonds formed between the carboxylate group of IL-4Rα Asp125 and the side chain amino group of IL-4 Gln78 and the guanidinium group of IL-4 Arg81. For the IL-4 variant T13D, the affinity to IL-4Rα D125A is decreased by a factor of 5; for IL-4 F82D, the affinity is reduced 8-fold compared to wild-type IL-4Rα (Table 3). Finally, the IL-4Rα variant Y127F was tested for binding to IL-4, T13D and F82D. The hydroxyl group of Tyr127 is involved in a hydrogen bond with Thr13 of IL-4. Substitution of IL-4Rα Tyr127 with a phenylalanine leads to only a small (2-fold) reduction in binding. A slightly larger reduction in binding affinity is observed for the IL-4 super-agonists T13D (4.5-fold) and F82D (2.5-fold).

Interestingly, none of the IL-4Rα variants showed a strong cooperative change in binding affinity for the IL-4 super-agonist T13D and F82D when compared to wild-type IL-4, suggesting that the additional interactions between T13D-F82D and IL-4Rα are not formed directly between Asp13 or Asp82 and any of the IL-4Rα side chains investigated here. The greatest difference in binding characteristics between wild-type IL-4 and the super-agonist T13D was observed for the IL-4Rα variant D67A. Since the side chain of IL-4Rα Asp67 is hydrogen-bonded to IL-4 Arg85, this suggests that the side chain of Arg85 is involved in different interactions in the ligand-receptor complexes of wild-type IL-4 and T13D. The differences in side chain orientation of Arg85 between the structures of free IL-4, T13D and F82D (Fig. 3a-c) might therefore also be present in the IL-4 ligand-receptor complexes.

### Homology modelling of IL-4 ligand-receptor interaction

Unfortunately, we have not succeeded so far in obtaining diffracting crystals for the ligand-receptor complexes comprising the IL-4 super-agonists T13D and F82D bound to the extracellular domain of IL-4Rα. In order to obtain insights into the possible interaction mechanism, we have modelled the interaction of T13D and F82D with IL-4Rα on the basis of the crystal structure of wild-type IL-4. The changes observed in the structures of the free IL-4 super-agonists T13D and F82D were transferred on to the complex structure by superimposing the free structures of the ligand on to the IL-4 bound to IL-4Rα. To obtain the model of the T13D-IL-4Rα complex, the coordinates of Asp13 (of T13D) and of Arg85 were used instead of the coordinates of the original residues Thr13 and Arg85 of wild-type IL-4. The same procedure was used for the complex of F82D-IL-4Rα. The amino acid exchanges did not cause bad van der Waals contacts, nor was the packing of the side chains in the interface impaired.

**Figure 4:**
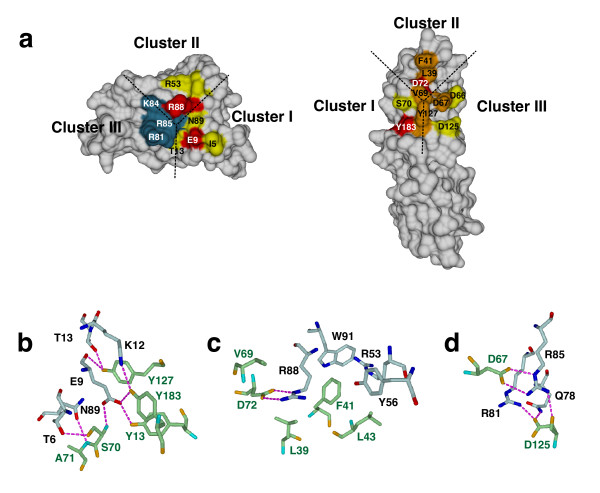
**Modular interface of the IL-4 – IL-4 receptor interaction**. (a) The interface between IL-4 and IL-4Rα consists of three clusters that contribute binding free energy independently of each other. Hydrogen bonding only occurs within one particular cluster but does not extend between two different clusters. Color coding represents the binding free energy that each residue contributes to the ligand-receptor interaction (red: ΔG = 3.5 kcal mol^-1^; orange: 3.5 kcal mol^-1 ^> ΔG = 1.7 kcal mol^-1^; yellow: 1.6 kcal mol^-1 ^> ΔG = 0.5 kcal mol^-1^; dark grey: 0.5 > ΔG > 0 kcal mol^-1^. (b) Cluster I is centred on Glu9 of IL-4, which is one of the two main binding determinants of the IL-4 – IL-4Rα interaction. (c) IL-4 Arg88 is the central residue in cluster II forming a bi-dentate saltbridge with IL-4Rα Asp72. (d) Cluster III consists of a hydrogen-bonding network comprising positively charged residues on the IL-4 interface (Arg81, Arg85) and negatively charged residues on the IL-4Rα epitope (Asp67, Asp125).

The structure of the complex of wild-type IL-4 bound to IL-4Rα shows that the ligand-receptor interface has a modular architecture [33], which, from analysis of the hydrogen bonding, can be described by three independently interacting clusters (Fig. 4a). Two of these three clusters include the so-called main binding determinants, Glu9 (IL-4) and Arg88 (IL-4), which contribute about 80% of the total binding energy. Cluster I is centred on Glu9 of IL-4, which makes several hydrogen bonds with Tyr13 (hydroxyl group), Ser70 (main chain amide) and Tyr183 (hydroxyl group) of IL-4Rα (Fig. 4b). Cluster II involves Arg88 of IL-4, which forms a bi-dentate salt bridge with Asp72 of IL-4Rα (Fig. 4c). Cluster III consists of the positively charged residues Arg81 and Arg85 of IL-4 and the negatively charged residues Asp66, Asp67 and Asp125 of IL-4Rα (Fig. 4d). Although the charged amino acids in Cluster III of the ligand are distributed in a highly complementary manner, these residues do not contribute to the binding affinity, as was shown by mutagenesis and BIAcore analysis [39]. Arg81 and Arg85 of IL-4 form several hydrogen bonds with residues of IL-4Rα; however, these interactions do not seem productive.

**Figure 5:**
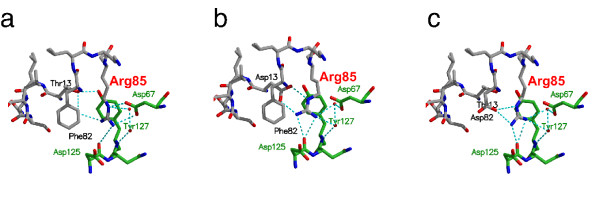
**Hydrogen bonds network in the IL-4 – IL-4 receptor interface**. (a) The hydrogen bond network in cluster III (stippled lines in cyan) between wild type IL-4 and IL-4Rα is shown. Arg85 of IL-4 is H-bonded to residues of IL-4Rα (Asp67 side chain, Asp125 main chain), but several of these H-bonds are mediated via solvent molecules. (b) Model for the interaction of the super-agonist IL-4 T13D with IL-4Rα. The "internal" H-bonds between Asp13 and Arg85 orient the side chain of Arg85 to yield intermolecular H-bonds to the IL-4Rα Asp125 main chain carbonyl. (c) A similar mechanism as in (b) can be drawn for the IL-4 F82D variant when an intramolecular H-bond between IL-4 Asp82 and Arg85 is assumed.

Modelling of the interaction of T13D bound to IL-4Rα suggests that the guanidinium group of Arg85 (T13D) now forms bi-dentate hydrogen bonds with the main chain carbonyl of the receptor Asp125 (Fig. 5b). In contrast to the hydrogen-bonding network of Arg85 in wild-type IL-4 (Fig. 5a), Arg85 in the complex of T13D:IL-4Rα is probably also fixed to Asp13 (T13D) via a bi-dentate salt bridge. This internal hydrogen bonding would result in fixation of the Arg85 side chain prior to complex formation. Therefore, the conformational entropy is not decreased for Arg85 in the formation of the T13D-IL-4Rα complex, whereas for wild-type IL-4 the side chain of Arg85 becomes ordered only upon binding to IL-4Rα. Consequently, the entropy cost for immobilizing this side chain neutralizes the energy release of its hydrogen bonding interactions.

The model of the F82D:IL-4Rα complex allows us to propose a stabilizing mechanism similar to that suggested for the complex of T13D and IL-4Rα (Fig. 5c). Here, too, the carboxylate group of Asp82 (F82D) forms a bi-dentate salt bridge with the guanidinium group of Arg85, thereby immobilizing the side chain of Arg85 in the ligand-receptor interface. The "head groups", i.e. the two imino groups, form two hydrogen-bonds with the main chain carbonyl of Asp125 (IL-4Rα), as also observed in the model of T13D bound to IL-4Rα (Fig. 5b). Hence the two amino acid changes in T13D and F82D lead to an identical change in the side chain conformation of Arg85 (ligand). This change of Arg85 subsequently transduces the additional stabilization/interaction observed for the two super-agonists. Since the additional interaction is mediated in both super-agonists by the same indirect mechanism via Arg85, this also explains why combination of two super-agonistic mutations did not lead to a further increase in binding affinity.

### A change in side chain conformation of IL-4 Arg85 leads to an increase in binding affinity

**Figure 6:**
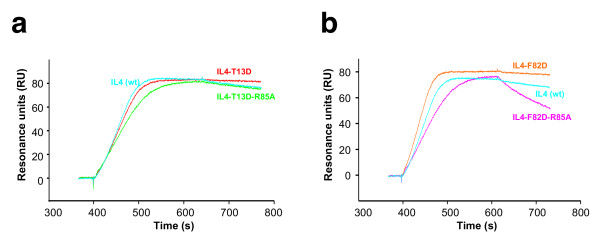
**Arg85 is involved in the binding mechanism of the IL-4 super-agonist T13D and F82D**. BIAcore sensorgrams of ligand-receptor interactions. Solutions of IL-4 variants (concentration 10 nM) were perfused over a sensor chip with immobilized IL4Rα_ECD_. (a) The variant IL4-T13D-R85A (green) shows a similar "fast" dissociation rate (*k*_off_) as wild-type IL-4 (blue); the binding kinetics of the super-agonist IL4-T13D are shown in red for comparison. (b) As for T13DR85A, the variant F82DR85A (magenta) exhibits a fast dissociation rate compared to the super-agonistic single amino acid variant IL4-F82D (orange). However, the dissociation is even faster than wild-type IL4 (blue).

We tested the interaction model proposed above by mutating Arg85 of IL-4 and the super-agonistic variants to alanine and measuring the residual binding affinity to IL-4Rα. If Arg85 is really involved in generating the additional stability/interaction, then, owing to cooperativity, mutation of this residue would have a greater effect in the super-agonistic variants (decrease in binding affinity) compared to wild-type IL-4. Changing Arg85 of IL-4 to alanine does not change the binding affinity for IL-4Rα dramatically (*K*_D _(R85A)/*K*_D _(wt IL-4) ~ 4.7-fold, thus ΔΔG = 1 kcal mol^-1^). This is in line with the observation that residues in Cluster III do not generally contribute to the overall binding (Fig. 4a, 6a). In particular, the dissociation rate constant is only increased by a factor less than 1.3, showing that the hydrogen-bonding network of Arg85 with IL-4Rα provides no free binding energy. In contrast, the affinity of the IL-4 variant T13D-R85A is decreased 6.5-fold and its dissociation rate reflects IL-4 wild-type-like binding kinetics (Fig. 6a). Since the hallmark of the super-agonistic variants was a clearly decreased dissociation rate constant (relative *k*_off _(T13D or F82D) = 0.3 – 0.4 *k*_off _(IL-4wt)), this confirms the above mechanism by which Arg85 is involved in generating the higher binding affinity. An even bigger effect is observed for the variant F82D-R85A (Fig. 6b). Here the equilibrium binding constant is increased almost 36-fold, mainly because of the increased dissociation rate (*k*_off _(F82D-R85A)/*k*_off _(F82D) ~ 9). To exclude the possibility that structural changes in addition to the removal of the Arg85 side chain play a role in the changes in binding stability, we determined the structures of the IL-4 variants R85A, T13D-R85A and F82D-R85A. No changes in the local structure around residues Thr/Asp13, Phe/Asp82 or Arg85 could be observed (data not shown).

### IL-13 utilizes the same main binding determinants for binding to IL-4Rα

**Figure 7:**
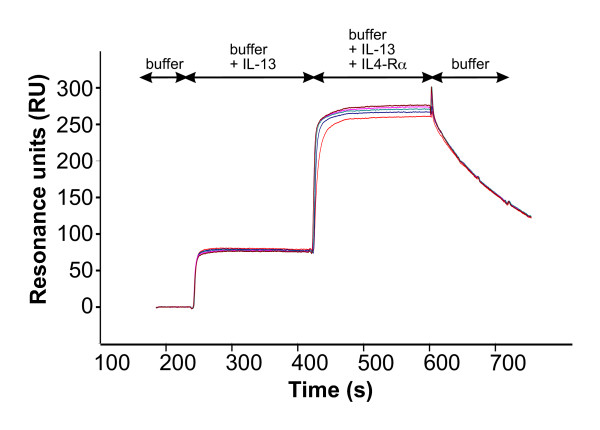
**BIAcore analysis of interaction between IL4-Rα and the IL-13/IL13-Rα1 complex**. BIAcore sensorgrams of the interaction between IL-4Rα and the binary complex of IL-13 and IL13-Rα1. IL-13 (1 μM) is perfused over a sensor chip with immobilized IL-13Rα1_ECD _to form a binary complex. After saturation of the immobilized IL13Rα1 chains with IL-13, IL4-Rα_ECD _(concentrations 1, 2.5, 5, 7.5, 10 μM) is perfused over the binary complex to measure the binding of the IL4-Rα chain to the complex of IL-13 and the IL13Rα1 chain. The apparent dissociation constant *K*_D _of approx. 150 nM for the binding of the IL4Ra chain to the binary complex was calculated by evaluating dose dependency of equilibrium binding.

IL-13 uses the same cellular receptor for signalling as IL-4, consisting of the IL-4Rα and IL-13Rα 1 subunits [40,41]. However, the binding mechanism is different and the order of the binding events is reversed. IL-13 binds first with moderate to high affinity to its IL-13Rα1 receptor chain (*K*_D _~ 25 nM) as determined from BIAcore experiments [42]. Then the second subunit is recruited into the complex. In contrast to IL-4, only one "low-affinity" chain, i.e. IL-4Rα, is able to bind to the complex (Fig. 1b,7). The apparent affinity (*K*_D _~ 80 nM) of the extracellular domain of IL-4Rα to the binary complex of IL-13 bound to IL-13Rα1 (Fig. 7) is rather high compared to the low apparent binding affinities observed for the interaction of the binary complex of IL-4:IL-4Rα with either γ_c _or IL-13Rα1 (*K*_D _~ 2 to 5 μM). The binding sites for IL-13Rα1 and IL-4Rα on IL-13 have been mapped by mutagenesis and functional assays [35,37]. The binding epitope for IL-13Rα1 is located on the C-terminal end of helix D of IL-13; the epitope for the IL-4Rα is located on the helices A and D. The two main binding determinants of IL-4 to IL-4Rα_ECD_, i.e. Glu9 and Arg88, map to the residues Glu11 and Arg64 of IL-13 (numbering according to the mature part of SWISS-PROT entry P35225) when the structures of IL-4 [24,26,27,31,38] and IL-13 [22,23] are superimposed. Both IL-13 variant proteins, E11A and R64A, were prepared and their binding properties for the ECDs of IL-13Rα1 and IL-4Rα were determined by BIAcore analysis. The binding affinities of both variants to IL-13Rα1 were unchanged compared to wild-type IL-13 (data not shown). Binding to IL-4Rα was measured by binding IL-13 first to immobilized IL-13Rα1_ECD _and then perfusing IL-4Rα_ECD _together with IL-13 over this binary complex. The apparent binding affinities were 35 μM and 200 μM for IL-13 E11A and IL-13 R64A, respectively. The dramatic loss in affinity clearly indicates the requirement of both residues for binding of IL-13 to IL-4Rα, which has also been shown previously in a more qualitative cell-based measurement [37]. Compared with the apparent affinity of wild-type IL-13, the affinity drops by factors of 230 and 1300 for IL-13 E11A and IL-13 R64A, showing that although the affinity of IL-13 for IL-4Rα is 1000-fold lower than that of IL-4 for IL-4Rα, both main binding determinants are conserved. Remarkably, IL-4Rα_ECD _is bound with a relatively high affinity (*K*_D _= 80 nM) to the binary complex of IL-13 and IL-13Rα1_ECD _compared to the low-affinity interaction (*K*_D _~ 2 μM) of IL-13Rα 1_ECD _to the binary complex of IL-4 and IL-4Rα_ECD_. Although this suggests that the affinities of IL-13 to the two receptor subunits IL-13Rα1_ECD _(*K*_D _~ 25 nM) and IL-4Rα_ECD _(*K*_D _~ 80 nM to IL-13-IL-13Rα1_ECD _complex) are of similar magnitude, the binding mechanism is still absolutely sequential, since IL-13 alone does not bind to IL-4Rα_ECD _(Fig. 1b). This clearly shows that the binding of IL-4Rα_ECD _to the binary complex comprising IL-13 and IL-13Rα1_ECD _is highly cooperative and probably involves a large receptor-receptor interface. The cooperative binding mechanism of the IL-13 ligand-receptor interaction is quite different from that of IL-4; here the overall binding affinity is dominated by the interaction of IL-4 with its high-affinity receptor subunit IL-4Rα; the low-affinity interactions do not add significantly to the overall binding free energy (Fig. 1a).

## Discussion

In this publication we provide a molecular mechanism by which the two IL-4 super-agonistic variants T13D and F82D bind to the extracellular domain of the IL-4Rα receptor with higher affinities than wild-type IL-4. The binding affinity for IL-4Rα of both variants is increased roughly 3-fold (Table 1). Structural analysis revealed that changes are limited to the very local environment around the site of mutation. A homology model of the ligand-receptor interaction was built, since attempts to crystallize complexes between the super-agonistic variants and the extracellular domain of the IL-4 receptor α have not so far been successful. The data clearly suggest that the concept of a modular protein-protein interface might allow binding affinity and specificity to be varied independently.

The "key-lock" principle used in the past to describe protein-protein interactions is based on the assumption that rigid molecules interact on the basis of surface geometry complementarities. This strict requirement would very probably result in monospecific interactions, i.e. only one molecule binds to another single molecule. However, cross-reactivity in antibody-antigen interactions showed early on that molecular recognition is much less specific. Nowadays many proteins, e.g. growth factors/-receptors, hormones, etc., or protein domains, have been shown to have multiple interaction partners that share limited sequence and possibly structural homology [43-48]. In the cytokine superfamily, the so-called redundant functions exhibited by many cytokines indicate the sharing of one or more receptor subunits, although sequence similarity is frequently below 25% within a superfamily [49]. This leads to the question how proteins generate binding affinity and binding specificity [50]. This question is difficult to answer, since what makes an interaction epitope is still largely unknown. Attempts to find common characteristics for protein-protein recognition sites have only been moderately successful, since the average binding epitope of a large compilation of protein-protein interfaces is almost indistinguishable from a regular protein surface [51]. It rather seems that there is a clear difference between the chemical and geometrical composition of a binding site and the regular surface if the binding versus non-binding sites are compared on a single protein. Nevertheless, computational analyses of protein-protein complexes have yielded some fruitful insights into the general characteristics of interaction interfaces. First, the degree of amino acid sequence conservation is increased within binding epitopes; secondly, the interfaces on two binding partners seem to be coupled in evolution, and thus can be used to predict binding epitopes and partners [52,53]. Furthermore, certain amino acids seem to be enriched in protein-protein interfaces (i.e. Arg, aromatic residues) [51]. A mechanism explaining how binding affinity is modulated on large protein epitopes was introduced by the concept of a binding hot spot by Cunningham and Wells [54,55]. Functional studies on human growth hormone showed that only a few residues within a rather large epitope generate the majority of the binding free energy; however, residues that are "non-productive" in terms of the generation of binding affinity might be important for specificity [56].

The IL-4/IL-13 receptor system represents an extreme model of ligand-receptor promiscuity, since both ligands, i.e. IL-4 and IL-13, can bind to the same cellular receptor consisting of the IL-4Rα and the IL-13Rα1 receptor subunits. Two further subunits, IL-13Rα2 and γ_c_, seem to be ligand-specific: the γ_c _subunit binds to IL-4 but not to IL-13 [40] while IL-13Rα2 binds exclusively to IL-13 [57]. Despite the use of an identical receptor, the binding mechanisms for the two ligands differ, and the binding affinities between the individual ligand and receptor proteins vary dramatically (by a factor of 200–1000). One ligand usually interacts with one receptor subunit (i.e. IL-4 with IL-4Rα, IL-13 with IL-13Rα1) with high affinity (*K*_D _~ 90 – 150pM for IL-4, ~ 20 – 30 nM for IL-13), but the other subunit is bound with lower affinity, usually in the high nM to μM range (e.g. IL-13Rα1 or γ_c _to the binary complex of IL-4 and IL-4Rα). This requires adjustment of the binding strength of the receptor subunits over a large bandwidth, but the specificity must remain high. The γ_c _receptor subunit is also shared among the cytokines IL-2, -4, -7, -9, -15 and -21 and can be bound by IL-4 instead of the IL-13Rα1 subunit [40,58]. However, this subunit always interacts as a low-affinity receptor chain with the binary complexes of the above-listed cytokines with their respective high-affinity receptor subunits, i.e. IL-2Rβ, IL-4Rα, IL-7Rα. Hence, the low specificity of the γ_c _receptor subunit might be a direct result of only low-affinity binding. The specificities of IL-4Rα and IL-13Rα1 for both ligands IL-4 and IL-13 are high; both receptor subunits only recognize IL-4 and IL-13 despite the low sequence identity between the two ligands and highly variable affinities. One possible mechanism by which different affinities for binding partners can be provided by a single protein involves the use of different epitopes or different subsets of residues within an overlapping epitope. Such mechanisms have been discussed for the binding of the common signal transducer chain gp130 in the IL-6 system [59]. Also, the γ_c _chain binds to IL-4 and IL-21 via such a mechanism [60]. However, these are different from the IL-4/IL-13 system, as their epitopes do not contain the modular architecture formed by several independent acting hot spots. The usage of one, two or all three hot spots in the IL-4/IL-4Rα interface allows the affinity to be scaled from high the nM to the pM range. Even if one of the hot spots is not "used" for binding, i.e. in terms of generating a significant contribution to the binding free energy, the non-contributor might be used to ensure specificity of interaction. According to this mechanism, we assume that all three hot spots are used for binding of the IL-4 super-agonist proteins, while only hot spots 1 and 2 are used in optimal form for wild-type IL-4 and hot spot 3 only contributes marginally. For binding of IL-4Rα to the binary complex of IL-13 and IL-13Rα1, only two hot spots are probably involved. For the interaction of different colicin endonucleases (E DNase) and the immunity proteins (Im), a similar mechanism termed "dual recognition" has been described [61,62]. Here, two epitopes/hot spots in close proximity (residing on different secondary structure elements) are used to generate binding affinity and specificity. One binding hot spot formed by three consecutive residues is used to generate a "basal" binding affinity, and the second epitope/hot spot adds additional affinity for cognate partners or is silent for non-cognate binding partners, as in our observation on the modular IL-4/IL-4Rα interface. The differences in affinities seem rather large – 10^11 ^to 10^5^-fold – owing to the very high affinities in this system. Even many non-cognate partners still bind with high affinity, e.g. *K*_D _~ 1 to 100 nM range, which is comparable to the high-affinity interactions in the IL-4/-13 system.

## Conclusion

Analyses of the modular architecture of the IL-4 – IL-4Rα interface yield a possible mechanism by which proteins might be able to generate binding affinity and specificity independently. Affinity and specificity were often considered to be linked, i.e. high specificity requires high affinity and vice versa. However, the binding affinities of IL-4 and IL-13 to IL-4Rα differ by a factor of more than 1000, but binding specificity remains absolutely high since the receptor subunit IL-4Rα binds exclusively to IL-4 and IL-13. Such an interface formed by several interaction clusters/hot spots of binding allows for a broad range of affinities by selecting how many of these interaction clusters contribute to the overall binding free energy. Non-contributing clusters will, however, still be important for the specificity of the interaction. Understanding how proteins generate affinity and specificity is especially important as more and more growth factor receptor families are found to show promiscuous binding to their respective ligands. However, this limited specificity is not always accompanied by low binding affinities. Knowledge of the details of the recognition mechanism will finally allow highly specific growth factors to be designed that are able to distinguish between different receptor combinations, as shown for the T-cell specific IL-4 antagonist IL-4R121E [63,64] or the IL-13 cytotoxin fusion used for treatment of several cancers [65,66].

## Methods

### Protein expression and purification

Human IL-4 was cloned into the expression vector pQE-80L (Qiagen) modified to carry a gene encoding kanamycin resistance; human IL-13 was cloned into the expression vector pET-28b (Novagen). Mutations in either IL-4 or IL-13 were introduced using the QuikChange (Stratagene) method. The expression vector constructs were transformed into *E. coli *BL21(DE3)Star (Stratagene) cells. For purification, cells from 4L cultures were lysed by sonication, and inclusion bodies were extracted and purified by extensive washing steps. Refolding of IL-4 was performed according to published protocols except that PBS buffer (phosphate buffered saline) pH 7.4 was used for dialysis [67]. Refolded IL-4 was purified by two cation exchange chromatography steps utilizing CM-Sepharose and High-Performance SP-Sepharose (Pharmacia), employing linear NaCl-gradients at pH 5.0 (25 mM acetate buffer) and pH 7.0 (25 mM phosphate buffer), respectively. Refolding of IL-13 followed the protocol published by Eisenmesser et al. [68]. Refolded IL-13 was purified by cation exchange chromatography using SP-Sepharose Fast Flow at pH 6.1 (25 mM phosphate buffer, 10 mM NaCl, 1 mM EDTA) and subsequently by RP-HPLC using a C4 Vydac column employing a linear gradient of 0–100% acetonitrile. The extracellular domain of the human IL-4 receptor α was expressed and purified from baculovirus infected Sf9 insect cell culture as described previously [19]. The receptor ectodomains of γ_c _and IL-21R were prepared as described [20,60].

### Proliferation assays

The bioactivities of IL-4 wild-type and variant proteins and of IL-13 were determined by measuring [^3^H]thymidine incorporation into the human premyeloid cell line TF-1 [69,70]. Cells were cultured in RPMI medium supplemented with 10% FBS and 2 ng ml^-1 ^recombinant GM-CSF. Cells were washed twice with PBS and seeded at a concentration of 5 × 10^3 ^cells/well in 96 well plates in RPMI medium without GM-CSF. Varying concentrations of IL-4 or IL-13 proteins (log_3 _dilutions starting at 1 μg ml^-1 ^as the highest concentration) were added, and the cells were cultured for 48 h. Tritiated thymidine (Amersham, 0.25 μCi/well) was added to each well 8 h before the plates were harvested using a Skatron cell harvester (Skatron Inc., USA). Filter mats were counted in a β plate counter. All experiments were performed in triplicate. Half-maximal responses of IL-4 and IL-4 super-agonist proteins in TF-1 cells were at approx. 10± 8 pM; for IL-13 the half-maximal response was observed at a concentration of 600± 200 pM.

### Crystallization of IL-4 and variants

Human IL-4 and variants thereof were further purified for crystallization by ammonium sulfate precipitation. Solid (NH_4_)_2_SO_4 _was added to a concentration of 1.2 M to solutions of approx. 10 – 15 mg ml^-1 ^protein. Precipitated impurities were removed by centrifugation, further (NH_4_)_2_SO_4 _was added to a final concentration of 2.5 M, and the mixture was kept on ice for 15 min. The mixture was centrifuged at 14.000 × g for 15 min at 4°C and the precipitated IL-4 was washed twice with 2.5 M (NH_4_)_2_SO_4_. The IL-4 was then dissolved in 1.2 M ammonium sulfate, pH 7.0, at a final protein concentration of 15 mg ml^-1^.

Crystals of IL-4 and variants were obtained by hanging drop vapor diffusion at room temperature using (NH_4_)_2_SO_4 _concentrations ranging from 1.9 to 2.4 M, and a pH range of 5.0 to 6.5. For measurements at 100 K, 25% glycerol was used as a cryoprotectant. High quality crystals grew from 2.2 M (NH_4_)_2_SO_4_, 0.1 M sodium acetate pH 5.2 and 25% glycerol using a protein concentration of 12 mg ml^-1^.

### Data collection

Data for IL-4 or its variants were each obtained from a single crystal at 100 K at different beamlines (X06SA PX at the Swiss Light Source, Switzerland, ID14-1 at the European Synchrotron Radiation Facility, Grenoble, France) or a home source (Rigaku MicroMax007 with Osmic VariMax mirror system). The data were processed and integrated using the software MOSFLM version 6.2.1, and scaling was performed using SCALA CCP4 version 4.2.1; a summary of the processing statistics of the various datasets is presented in Table 2. To test for possible bias introduced by the model structure IL-4 (PDB entry 1HIK) used for data interpretation by the molecular replacement method, we also collected a dataset of a SeMet-labeled IL-4 variant F82D at three wavelengths (BW7A, EMBL DESY, Hamburg, Germany) (see Table 4). Structures of the variant F82D refined by the MAD approach and molecular replacement were identical, showing that the model structure 1HIK used as the start structure for refinement did not bias the results of the individual variant structures.

**Table 4:** MAD data set for IL-4 variant F82D

Crystal	F82D		
Space group Cell constants	P4_1_2_1_2 a = b = 91.304 Å, c = 45.932 Å α = β = γ = 90°		
	Se-Met (λ1)	Se-Met (λ2)	Se-Met (λ3)
Wavelength	0.9844 Å	0.9803 Å	0.9078 Å
Resolution (Å)^1^	25.0 – 2.15 Å (2.27 – 2.15 Å)	25.0 – 2.15 Å (2.27 – 2.15 Å)	25.0 – 2.15 Å (2.27 – 2.15 Å)
	λ1 (inflection)	λ2 (peak)	λ3 (remote high)
Number of measured reflections	51335 (5658)	54273 (5689)	50834 (5624)
Number of unique reflections	10758 (1410)	10721 (1413)	10640 (1384)
Completeness	97.0 (90.6)	97.3 (91.2)	96.5 (89.2)
Multiplicity	4.8 (4.0)	5.1 (4.0)	4.8 (4.1)
R_sym _for all reflections^2^	5.6 (13.8) %	6.0 (12.1) %	4.7 (10.3) %
Intensity/σ	18.1 (7.4)	18.2 (8.0)	20.1 (11.0)
Phasing			
R_cullis _(a/c)^3^	0.689/0.533	0.725/0.651	-
R_Kraut_^4^	0.035	0.034	0.028
Phasing Power (a/c)^5^	1.60/1.90	1.42/1.65	0.93/1.09
Figure of merit^6^	0.55/0.79 (after DM)		

^1 ^number in parentheses indicate highest resolution shell.

^2 ^R_sym _= ∑_hkl_|I_hkl _– <I_hkl_>|∑_hkl_<I_hkl_> where <I_hkl_> is the mean intensity of symmetry-related observations of a unique reflection

^3 ^R_cullis _= <phase-integrated lack of closure>/<F_PH _– F_P_>

^4 ^R_Kraut _= ∑ |(|F_P_+F_H_|)-|F_PH_|)|/∑|F_P_|

^5 ^Phasing power = (√|F_H_^2^|)/(√|lack-of-closure^2^|), (a/c) denote acentric and centric reflections

^6 ^figure of merit is given before and after solvent density modification

### Structure analysis

The structures of IL-4 and the variants investigated in this study were refined using the lower resolution structure (PDB entry 1HIK) as a start model. To minimize possible bias through the start model structure, especially for the ill-defined loop regions, MAD phasing was applied to the IL-4 variant F82D. IL-4 (as well as the variant F82D) contains a single Met residue. The Seleno-Met site was determined and refined using the protocols supplied with the program CNS using a dataset measured at three wavelengths (inflection, peak and remote). The resulting electron density map was used to rebuild the loop regions between the first α-helix α_A _and the first short β-strand β_1 _(Glu19 to Cys24) as well as the long loops between β-strand β_1 _and helix α_B _(Lys37 to Glu41) and helix α_c _and the second β-strand β_2 _(Ser98 to Glu103). The resulting "improved" model was then used for interpretation of the diffraction data of the individual IL-4 proteins. The program REFMAC5 was used for subsequent refinement, followed by manual rebuilding of the models using the software QUANTA2000 (Accelrys Inc.). One TLS group was defined for the complete IL-4 molecule to account for anisotropy in the data. The progress of refinement was monitored by cross-validation using a test data set comprising 5% of the reflections. In the final refinement, *F*_obs _– *F*_calc _difference electron density maps were used to identify water molecules as well as sulfate ions resulting from the high concentration of the ammonium sulfate precipitant. The final conventional and free R-factors for each model are presented in Table 2.

### Interaction analysis using BIAcore

Interaction analysis was performed using a BIAcore 2000 system. (Pharmacia Biosensor). All experiments were carried out at 25°C at a flow rate of 50 μl min^-1 ^in HBS running buffer (10 mM Hepes, pH 7.4, 150 mM NaCl, 3.4 mM EDTA, 0.005% surfactant P20). The extracellular domain of the IL-4 receptor α-chain (IL-4Rα_ECD_) and variants thereof were biotinylated and immobilized to a streptavidin-coated sensor chip CM5 at a density of 50–120 RU [19,39]. To determine the kinetic rate constants, sensor chips with a low density of IL4Rα_ECD _were used to minimize rebinding effects. The IL-4 receptor proteins were prepared and analyzed under conditions as described [19,39]. Interaction with IL-4 proteins was measured after regeneration of the chip surface with 4 M magnesium chloride. The sensorgrams were evaluated using the software BIAevaluation version 2.0, assuming a 1:1 interaction. Bulk face effects were corrected by subtracting the control flow cell (FC1) from all sensorgrams; non-specific binding was negligible. The analysis yielded kinetic rate constants for complex formation (*k*_on_) and dissociation (*k*_off_). The latter were evaluated at near saturating concentrations of analyte during the first 10s of complex dissociation to avoid any effects of the rate constant *k*_off _due to rebinding of the dissociating ligand. The apparent binding constant of each variant was obtained using six analyte concentrations (2 – 20 nM). Standard deviations were deduced from 18 independent measurements. Apparent dissociation constants *K*_D _were either calculated as *K*_D _= *k*_off_/*k*_on _or by evaluating the dose dependency of equilibrium binding. Mean values together with the mean standard deviation are presented in Table 1. Binding of IL-4 and IL-13 ligand proteins to their low-affinity receptor subunits (IL-13Rα1, γ_c _and IL-4Rα) were analyzed by a COINJECT experiment [20]. IL-13 protein (concentration 1 μM) was perfused over a CM5 chip surface coated with the extracellular domain of the IL-13 receptor α1-chain to saturate any binding sites fully. Different concentrations (1 – 10 μM in the case of wild-type IL-13 and 5 – 100 μM in the case of IL13-E11A and IL13-R64A) of IL-4Rα_ECD _were then perfused together with IL-13 ligand by the COINJECT procedure of the BIAcore2000 system. A similar setup was used to measure the low-affinity binding of IL-13Rα1 and γ_c _to IL-4 and its variants. Binding affinities were deduced from these binding experiments by evaluating the dose dependency of equilibrium binding (IL-13 variants) or directly from the binding kinetics (IL-13 wild-type). Binding constants derived from analysis of equilibrium binding are presented in Table 1.

## Authors' contributions

M. Kraich and M. Klein performed the mutagenesis, protein expression and purification and crystallization of the IL-4 ligand and receptor proteins. E.P. and H.H. participated in the purification of the IL-13 ligand and receptor proteins. J.N. participated in the analysis of the interaction analysis data, W.S. performed the BIAcore measurements and analyses; T.D.M conceived the study and participated in all stages of the work. All authors read and approved the final manuscript.
